# Analysis of Pharmaceutical Demand in the Region for Chronic Medicine Users Using National Health Insurance Data: Examination for Disaster Preparedness in Hakui City, Ishikawa Prefecture

**DOI:** 10.3390/healthcare11233029

**Published:** 2023-11-23

**Authors:** Yuta Moriwaki, Yuma Morisaki, Shigehiro Karashima, Makoto Fujiu

**Affiliations:** 1 Division of Geosciences and Civil Engineering, Graduate School of Natural Science and Technology, Kanazawa University, Kanazawa 920-1192, Japan; y.moriwaki@stu.kanazawa-u.ac.jp; 2 Faculty of Transdisciplinary Sciences for Innovation, Institute of Transdisciplinary Sciences for Innovation, Kanazawa University, Kanazawa 920-1192, Japan; morisaki@staff.kanazawa-u.ac.jp; 3 Institute of Liberal Arts and Science, Kanazawa University, Kanazawa 920-1192, Japan; skarashima@staff.kanazawa-u.ac.jp

**Keywords:** medical big data, medical support, pharmaceutical demand, earthquake disaster

## Abstract

When large earthquakes occur over wide areas, in addition to damage to medical facilities, the disaster response capabilities of local governments are severely compromised. There is a very high possibility that the supply–demand balance of medicines will collapse within the disaster area, and that appropriate supplies of medicines will not be provided to disaster victims. Therefore, it is important to estimate in advance the quantity of pharmaceuticals that may be needed during disasters. In this study, the purpose is to clarify the quantity and quality of pharmaceuticals used by chronically ill patients by using Japanese National Health Insurance data regarding the issues mentioned above. The methodology used was to extract the status of pharmaceutical prescriptions based on receipt information from National Health Insurance data for Hakui, Ishikawa Prefecture, a small regional city in Japan, as the analysis target area. Through the analysis in this study, the quantity and quality of medicines supplied to chronically ill patients in Hakui, Ishikawa Prefecture, were clarified on a town-by-town basis.

## 1. Introduction

### 1.1. Background of This Study

When a large-scale disaster occurs, regional medical supply capacity decreases owing to damage to medical institutions, pharmacies, etc. Regional medical needs also increase owing to injuries caused by disasters, loss of pharmaceuticals, and worsening of mental and physical injuries and illnesses. Consequently, large medical needs for regional medical resources are generated, often leading to a major shortage of medical resources. Thus, the limited medical resources must be utilized effectively [1].

Regarding medical responses within the disaster area during a disaster, the priority for treatment and transportation of victims is determined by triaging the victims by quickly assessing the urgency and severity of the injury or illness. This results in appropriate medical resources being allocated to patients who require medical care and who can be expected to benefit from it in the event of a disaster. Meanwhile, medical response to patients with mild symptoms or patients for whom emergency treatment is difficult may be postponed, which results in circumstances wherein medical resources may not be immediately available [2].

Among such patients, those with chronic diseases and who use pharmaceuticals on a chronic basis are often relegated in terms of the prioritization of medical response in the above-mentioned triage evaluations, regardless of the fact that continuous use of pharmaceuticals is needed to manage their diseases. In particular, during disaster situations, the risk of worsening a patient’s condition increases owing to anxiety and deterioration of the living environment [3]. Furthermore, because of the deterioration of regional medical functions and the shortage of medical resources, it may not be possible to implement prompt medical responses when the patient’s condition suddenly changes.

Further, during a disaster, a large quantity of pharmaceuticals is externally supplied to disaster base hospitals and pharmacies in the affected area to provide support to pharmaceutical users. However, issues such as a large amount of time needed for the necessary pharmaceuticals to reach the patient remain, owing to reasons such as difficulties in sorting large quantities of pharmaceuticals because of personnel shortages and the supply of pharmaceuticals that differ from the needs within the affected area [4].

An example of a large-scale earthquake disaster that caused pharmaceutical problems in the past is the Great East Japan Earthquake in 2011. During the Great East Japan Earthquake, pharmaceuticals for emergency medical care were brought in by disaster medical assistance teams (DMATs) [5] to provide medical support immediately after the occurrence of the disaster. However, it was reported that in disaster-affected areas, patients with chronic diseases had a high pharmaceutical demand, and that a mismatch between supply and demand for pharmaceuticals resulted in a shortage of daily pharmaceuticals. It has also been reported that there were problems with the supply of pharmaceuticals owing to the long time required to sort the large quantities of pharmaceuticals that were brought in as relief supplies [6].

As mentioned above, when pharmaceutical users experience delays in the medical response or are forced to abruptly discontinue pharmaceutical treatment, it is possible that their condition may worsen. Thus, a prompt pharmaceutical support system for pharmaceutical users is needed in the event of a disaster.

Current pharmaceutical supply systems during disasters include strengthening collaboration within and outside the region by establishing disaster base hospitals and pharmacies in each prefecture, building a collaborative system with medical organizations such as DMATs [7], and listing of pharmaceuticals that may be needed in the disaster area in the event of a disaster by the Japan Medical Association [8]. However, in the current pharmaceutical supply system during disasters, as mentioned above, doctors and pharmacists travel to the affected area after the occurrence of a disaster and provide supply after understanding the pharmaceutical needs of the region. Therefore, it often takes time for the pharmaceutical supply to be delivered to the disaster-affected area.

Additionally, in the future, Japan is expected to face severe damage from large-scale disasters such as the Nankai Trough megathrust earthquake, which is expected to cause strong shaking and high tsunamis across a wide area from Kanto to Kyushu, and an earthquake directly underneath Tokyo, which is feared to affect the central functions of the capital [9]. Under such large-scale disaster situations, the current pharmaceutical supply system during disasters cannot facilitate the supply of pharmaceuticals in time for a majority of chronic pharmaceutical users, which results in a worsening of their condition or an endangering of their lives.

Based on the above results, it can be said that establishing a medical system that can quickly grasp the pharmaceutical needs of chronic pharmaceutical users in the event of a disaster and supply pharmaceuticals according to the needs of the disaster-affected area are urgent issues.

### 1.2. Purpose of This Study

A countermeasure to the above-mentioned issue is to examine in advance the pharmaceutical prescription status of chronic pharmaceutical users during normal times, thereby rendering it possible to provide support according to the pharmaceutical needs in a disaster-affected area. Additionally, as this is based on the pharmaceutical prescription status during normal times, this can be used to consider what pharmaceuticals should be stocked in the region in preparation for a disaster.

The purpose of this study was to clarify the types of pharmaceuticals prescribed and the number of people using them in Hakui, Ishikawa Prefecture, based on the status of pharmaceutical prescriptions during normal times, as a preliminary examination to provide pharmaceutical support according to the needs in the affected area during disasters. To that end, this study aimed to clarify the types of pharmaceuticals prescribed in a region and the number of users based on the pharmaceutical prescription status in normal times. In addition, the number of people using pharmaceuticals who would evacuate to shelters in Hakui during earthquake disaster was estimated, and the pharmaceutical needs of the shelters were examined.

**Figure 1:**
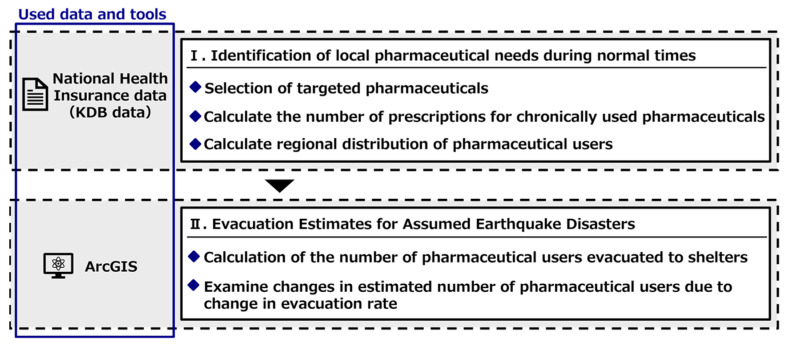
Flow of this study.

The flow of this study is shown in Figure 1. First, the “National Health Insurance Data” (“KDB data”) were used to examine the types of pharmaceuticals chronically used and the number of users in each town or city under normal circumstances. Thereafter, we estimated the number of pharmaceutical users who would evacuate to each evacuation shelter in the event of an earthquake disaster in Hakui City to clarify the number of pharmaceutical users at each evacuation shelter in the event of an earthquake disaster. The availability of evacuation depends on the actual scale of damage and the willingness of individuals to evacuate during disasters. Therefore, the number of people using pharmaceuticals in evacuation centers was clarified when the evacuation rate was varied from 0 to 100%.

## 2. Related Work

In conducting this study, we summarized previous research and positioned the present study based on the following two perspectives: research on pharmaceutical supply and demand during disasters and research on improving the efficiency of medical support during disasters.

### 2.1. Research on Pharmaceutical Supply and Demand during Disasters

Inaba et al. [10] summarized the contents of disaster prescriptions filled at temporary dispensaries and mobile pharmacies in Minamiaso Village, Aso District, Kumamoto Prefecture, during the 2016 Kumamoto earthquake. They determined the actual usage of dispensaries during disasters. Their results revealed that temporary dispensaries were used for 13 days and they primarily prescribed painkillers, cold medicines, gastrointestinal medicines, antihypertensive drugs, and sleeping pills. In contrast, mobile pharmacies were operational for 43 days and they primarily prescribed pharmaceuticals for chronic illnesses. Further, a questionnaire survey of disaster relief pharmacists showed that one of the issues during disaster relief activities was the large quantity of medicines that were discharged during relief activities.

Shiba et al. [11] determined the number of patients and pharmaceutical prescriptions from medical records and prescriptions obtained during mobile medical visits during the Great East Japan Earthquake to clarify the actual circumstances of long-term disaster medical support. The results showed that the number of patients decreased from a peak of 166 patient/day at one week after the start of support on 26 March, with 24 patients/day after 11 April. The number of pharmaceutical prescriptions was highest for cold medicine, followed by antihypertensive and antiallergy medicines, in descending order. Further, the amount of pharmaceutical usage decreased over time for cold medicine and antihypertensive medicine; however, antiallergy medicines continued to be prescribed.

Anami et al. [12] conducted an analysis of the characteristics of disaster prescriptions issued in Minamiaso Village, Aso District, Kumamoto Prefecture, and the prescription deficiencies during the Kumamoto earthquake disaster. The results showed that 41.1% of prescriptions were for long-term use (medications for hypertension, dyslipidemia, and diabetes), suggesting the need for a pharmaceutical supply system that considered chronic illnesses during disasters. Moreover, regarding the prescription deficiencies, several deficiencies in usage and storage were observed, which suggested the need for improvements to the format and operation method of disaster prescriptions.

There are also studies examining the supply of medicines for disasters occurring outside of Japan. Basu et al. [13] conducted a grouping of medicines needed during disaster using data available from the social networking service WhatsApp for the Nepal earthquake in 2015. Rossi et al. [14] used administrative data to identify psychotropic drug needs during an earthquake in Italy. Gholamreza et al. [15] investigated the most used medicines in the 6 months after the Bam earthquake in December 2003. In addition, Michael et al. [16] evaluated the relationship between actual pharmaceutical demand and the supply of medical relief drugs for 18,000 evacuees relocated to San Antonio, Texas, after Hurricane Katrina hit the Gulf of Mexico in August 2005.

### 2.2. Research on Improving the Efficiency of Medical Support during Disasters

Shikamura et al. [17] sought to effectively utilize general pharmaceuticals that require guidance and are supplied as relief supplies to disaster-affected areas (“OTC pharmaceuticals”) by examining the ideal form of OTC pharmaceuticals and creating a list of pharmaceuticals that can be used in times of disaster. Consequently, they suggested that OTC pharmaceuticals were more useful than medical pharmaceuticals because of the advantages while taking those pharmaceuticals, such as the ability to take them without water; however, there were differences in efficacy, effectiveness, applicable target age, and drug information.

Ishiwata et al. [18] sought to efficiently utilize the pharmaceuticals existing in disaster-affected areas by sharing pharmaceutical information owned by support teams and improving the convenience of pharmaceutical management during disasters in order to construct a disaster pharmaceutical management system. They built a new system on the cloud that enabled information exchange with relief stations and collection points via the Internet. This facilitated the sharing of pharmaceutical inventory information with relief stations and collection points in the disaster area. It was also suggested that the problems faced by support teams, such as pharmaceutical requests and geographical conditions, could be alleviated by creating a pharmaceutical inventory list of relief stations and confirming the locations of relief stations on a map.

Nakasako et al. [19] used the 56 days of medical support experience at a relief station during the Great East Japan Earthquake to analyze pharmaceutical prescription trends and information posted on group pages among medical professionals, and examined the changes in pharmaceutical demand and the construction of a support system at the same relief station during a disaster. They reported that the pharmaceutical demand became widespread from the sub-acute stage of the disaster. Further, smooth logistical support was possible by sharing information using group pages, which suggested that continuous support from the same medical institution was effective for efficient medical support.

Studies have also been conducted outside of Japan to examine the efficiency of pharmaceutical supply during disasters. Chen and Wanbon [20] classified pharmaceuticals based on three years of pharmaceutical purchase history and reviews by physicians and clinical pharmacy experts for a Vancouver Island hospital. This study estimated the amount of medicines that might be needed in a disaster based on the purchase history of medicines in normal times, which is similar to our study. There also exists research on approaches to develop mathematical modeling for an effective and efficient pharmaceutical supply chain [21,22].

### 2.3. Positioning of This Study

This study aims to clarify the pharmaceutical needs in the affected areas in advance of earthquake disasters and to obtain knowledge for prompt pharmaceutical support during disasters by examining the prescription status of pharmaceuticals in the area under normal circumstances.

Many studies on the supply of medicines during disasters have been based on post-event evaluations of past disasters. In Japan, the research field of this paper, many studies have been conducted mainly on the Tohoku-Pacific Ocean earthquake and the Kumamoto earthquake. Most of the data were obtained through interviews with doctors and questionnaires, and thus have a strong aspect of ex-post evaluation. Outside of Japan, many studies have investigated problems in the supply of medicines following disasters in various parts of the world. Various data were used, including SNS data among medical groups and prescription data. These studies also have a strong aspect of ex-post evaluation of the supply of medicines in past disasters. On the other hand, Rossi et al. [14] utilize Italian administrative data. In this respect, this study is similar in that it also uses KDB data obtained from the public administration. However, Rossi et al. [14] focused only on psychotropic drugs, which limited the types of medicines. Our study examines the amount of medicines needed in times of disaster by estimating the supply of medicines in normal times, utilizing medical big data provided by the local government. Furthermore, this study differs from other studies in that it targets multiple medicines, rather than only a few medicines as in previous studies. Multiple medicines are required at the same time during disaster. Therefore, this study, which examines multiple types of pharmaceutical needs, is novel as a study of pharmaceutical supply during a disaster. Furthermore, Chen and Wanbon [20] grouped the medicines needed in times of disaster, which is not a study of past disasters, but a perspective of preparedness in times of peace. In this regard, the study is similar to ours. However, it differs in that it targets only one medical facility. Our study differs in that it identifies the pharmaceutical needs of citizens within a single administrative area. As described above, this study differs significantly from other previous studies in that it extracts the demand for multiple types of medicines for a single administrative region. We believe that this study will provide new insights into the estimation of pharmaceutical demand during disasters.

## 3. Hakui City, Ishikawa Prefecture

### 3.1. Overview of Hakui City, Ishikawa Prefecture

**Figure 2:**
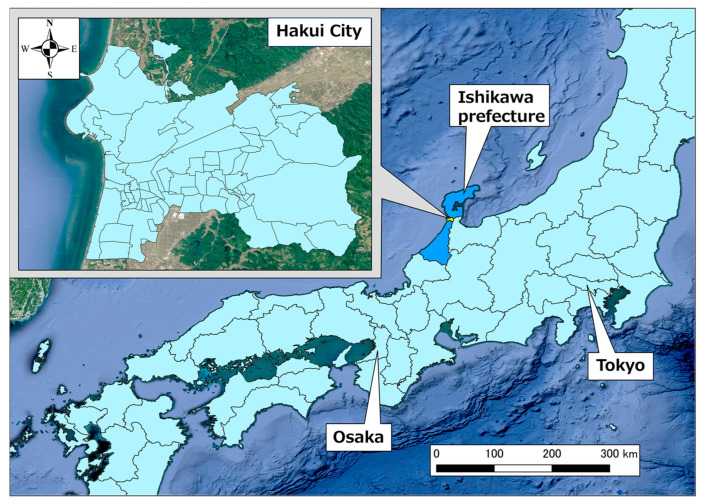
Position of Hakui City, Ishikawa Prefecture.

Figure 2 shows the location of Hakui City, Ishikawa Prefecture, which was the target area for this study. Hakui City is located on the west side of the base of the Noto Peninsula, and the city area is approximately 11 km from north to south and east to west. The area is sandwiched between the Sea of Japan to the west and Goshigamine and the mountains and seas to the east, with an urban area in the southwest of Hakui City, Ochigata, rural areas in the center, and mountainous areas in the east and north. The total population as of April 2020 was 20,004 people, of which 40.9% were aged 65 years or older [23]. There are also a total of 67 town districts in Hakui City.

### 3.2. Estimated Earthquake Disaster in Hakui City

Currently, in Hakui City, according to the “earthquake damage estimation survey” conducted by Ishikawa Prefecture, an earthquake with epicenters in the four fault zones of Daishoji Temple, Kaga Plain, Ochigata, and the northern coast of the Noto Peninsula are predicted to occur. The damage prediction results for each earthquake show that among the four earthquakes mentioned above, the earthquake caused by the Ochigata fault zone could result in the greatest damage, and in terms of human damage, it is predicted that there would be 21 fatalities, 595 injuries, and 365 people in need of rescue, with the number of evacuees expected at 5947 (approximately 29.7% of the population) [24].

**Figure 3:**
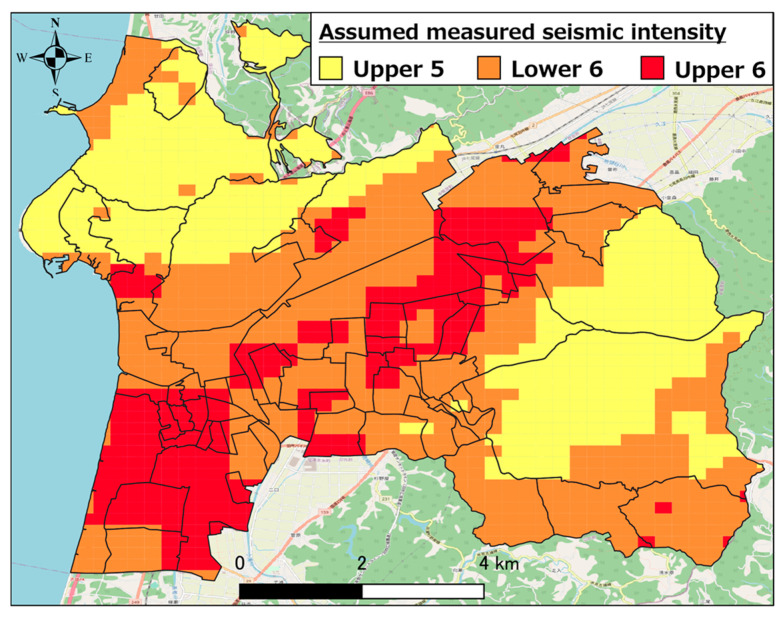
Estimated seismic intensity distribution when Ochigata fault zone is epicenter.

Figure 3 shows the assumed seismic intensity distribution [25] in Hakui City when the Ochigata fault zone is the epicenter. As evident, shaking with a seismic intensity of 5 lower or stronger can occur throughout Hakui City. Shaking with a seismic intensity of 6 upper to 6 lower is expected to occur in the southwestern part of Hakui City.

## 4. Overview of KDB Data and Examination of the Number of Chronic Drug Users in the Region

### 4.1. Overview of KDB Data

In this study, we used KDB data from Hakui City to clarify the pharmaceutical prescription situation within the region during normal times. The KDB data stores information on medical care, health checkups, and nursing care for people enrolled in the National Health Insurance, one of Japan’s medical insurance plans. The data started to be accumulated in Japan in April 2012, and individual data are stored on a monthly basis. All local governments in Japan must have this data, and it is managed in a common format. Originally, the data were used by local governments for effective data health planning, and they have not been opened to the public for academic purposes. However, the authors and Hakui city in Ishikawa Prefecture have a strong research partnership, so the data can be used for academic purposes. This information is accumulated every month. In addition, each data item has an identification number that is written on an individual basis, rendering it possible to link each piece of information on an individual basis. The coverage rate of KDB data in Hakui City was approximately 57.2% among those aged 40–74 years for National Health Insurance coverage, and 96.5% among those aged 75 years and older for elderly medical insurance.

In this study, among the KDB data for Hakui, the “insured person ledger” and the “medical abstract” were used to examine the types of pharmaceuticals chronically used in the community and the number of users.

The main information recorded in each data is the “insured person register”, which includes National Health Insurance enrollment information and personal information such as age, gender, and address. In addition, “medical summary” includes information such as the year and month of medical treatment and the prescribed pharmaceuticals if the patient received medical treatment at a hospital.

Further, the KDB data usage period was set at three months (September–November 2020). This was because the maximum prescription period for pharmaceuticals was 90 days, and patients using pharmaceuticals generally visit the hospital at least once within three months, including for follow-up monitoring of their condition. In the three months of KDB data, each individual has at least one prescription history for pharmaceuticals. By deleting duplicates of the same history, it is possible to obtain a comprehensive examining of the pharmaceuticals prescribed in Hakui.

Furthermore, Kanazawa University, with which the authors are affiliated, has concluded a comprehensive partnership agreement with Hakui City. Thus, the use of KDB data has been achieved. The KDB data used in this study were provided by Hakui City, and permission to use it for research purposes was obtained. Additionally, approval was obtained from the ethics review committee of our university (approval number: 2018-129 (053)).

### 4.2. Selection of Target Pharmaceuticals

In this study, KDB data are used to select chronic users of pharmaceuticals, and the extraction flow is shown below.

1. Based on discussions with doctors, selection of medicines that are chronically needed in times of disaster was conducted (Table 1). We selected target pharmaceuticals from the following two perspectives: “pharmaceuticals that are prescribed for illnesses that worsen individual conditions when not used” and “non-pharmaceuticals that require professional judgment when used.” Selection is made using the “Drug Classification (Subdivision Item)” [26], which is described in the KDB data. This is a list that identifies the type of drug prescribed.
2. Injectable drugs, topical drugs, and other treatment drugs are excluded from the selected drugs.
3. Selected drugs are extracted from the “drug effect classification (subdivision items)” listed in the KDB data. Since the KDB data are stored in Excel format, we extracted the drugs by filtering the selected drug effect classifications.

An example of the KDB data used in this study is shown in Table 2. IDs with confidential personal information and the types of drugs prescribed are accumulated. This part is used to calculate the number of persons who take pharmaceuticals in this analysis.

### 4.3. Examining the Status of Pharmaceutical Prescriptions in Hakui

**Figure 4:**
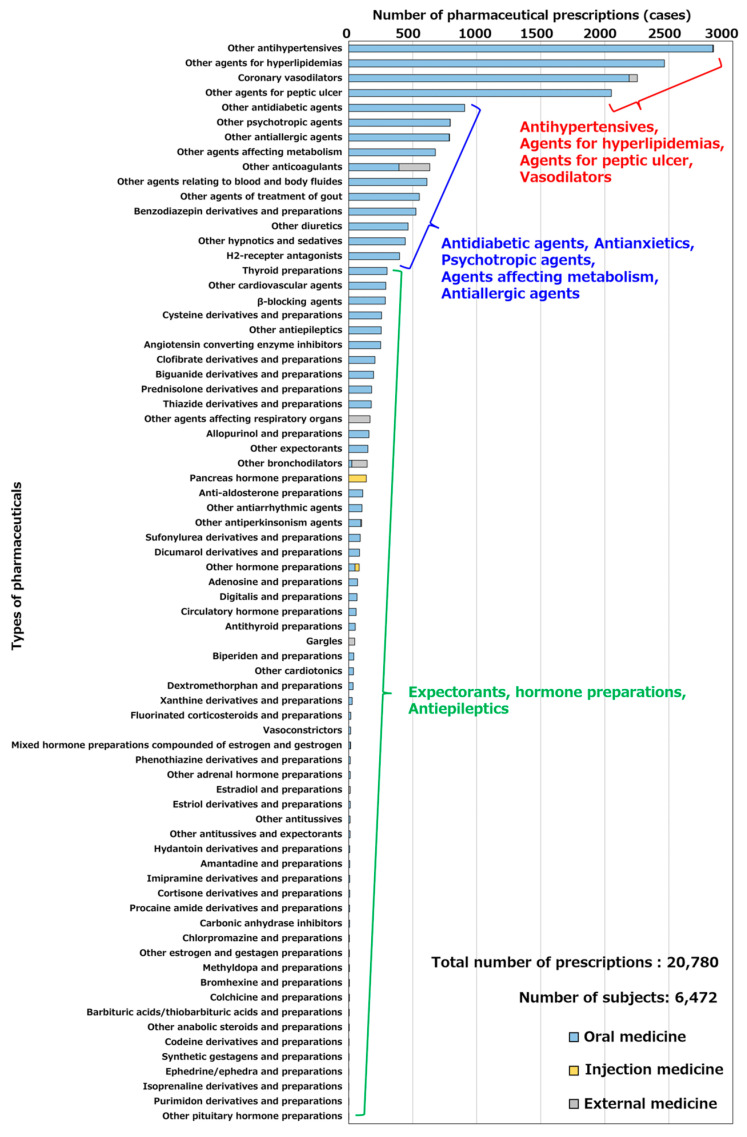
Number of prescriptions for each pharmaceutical in Hakui City.

The chronically used pharmaceuticals selected in Section 4.2 were extracted from the KDB data, and the number of prescriptions for each pharmaceutical was calculated. Figure 4 shows the number of prescriptions for each pharmaceutical in Hakui City. Based on the extraction flow described above, the number of people chronically taking drugs in Hakui, Ishikawa Prefecture, was calculated to be 6477 by November 2020. The chronically used pharmaceuticals in Hakui were other antihypertensives (2849 cases), other agents for hyperlipidemia treatment (2465 cases), and coronary vasodilators (2255 cases), and the number of prescriptions for lifestyle-related diseases such as hypertension and hyperlipidemia was particularly high. Other antidiabetic agents (906 prescriptions), other psychoneurotic agents (793 prescriptions), and other allergic agents (788 prescriptions) were prescribed more frequently. In contrast, fewer prescriptions were made for other antiepileptics (254 prescriptions), other expectorants (150 prescriptions), and hormonal agents.

Further, we calculated the regional distribution of the top three types of pharmaceutical users (other antihypertensive agents, other hyperlipidemic agents, and coronary vasodilators) in Hakui City as an example of the regional distribution of individuals using the target pharmaceuticals.

**Figure 5:**
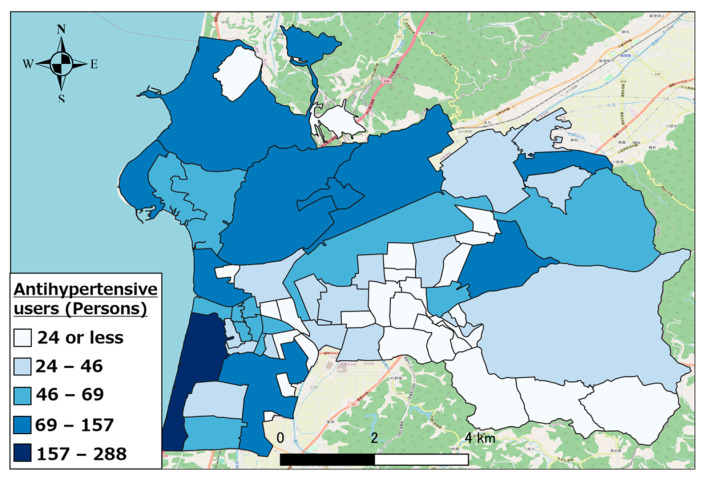
Distribution of other antihypertensive agent users.

**Figure 6:**
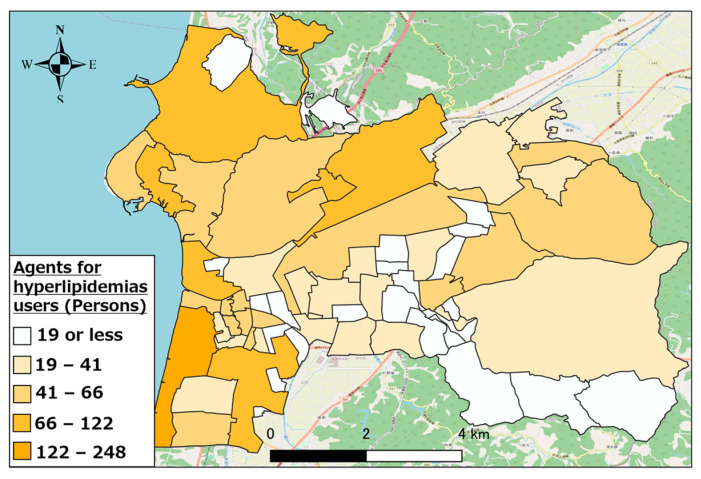
Distribution of other hyperlipidemic agent users.

**Figure 7:**
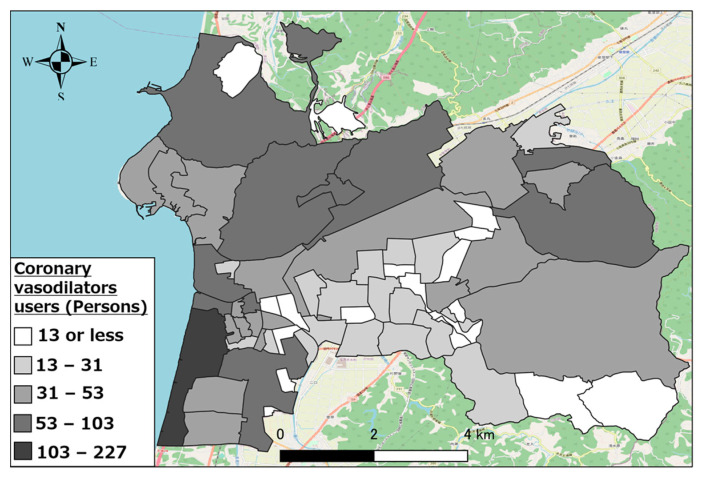
Distribution of coronary vasodilator users.

Figure 5, Figure 6 and Figure 7 show the distributions of other antihypertensives users, other agents for hyperlipidemia agents users, and coronary vasodilators users, respectively. As evident, there were numerous pharmaceutical users of each type in the urban area of western Hakui City. In contrast, there were fewer users of each type of pharmaceutical in the mountainous southeastern town of Hakui City.

## 5. Estimation of Pharmaceutical Needs at Evacuation Shelters in Hakui City

### 5.1. Method for Estimating the Number of Pharmaceutical Users Who Evacuate to Evacuation Shelters

In the event of a disaster, regional disaster base pharmacies that are established by prefectural pharmacist associations play a central role in providing pharmaceutical support within disaster-affected areas. Disaster base pharmacies stockpile pharmaceuticals during normal times in preparation for disasters and collect externally-supplied pharmaceuticals from outside the affected area in the event of a disaster. The flow of pharmaceutical supply within a disaster-affected area during a disaster is as follows: pharmaceuticals are collected at disaster base pharmacies, distributed to evacuation shelters and relief stations in the disaster-affected area, and then supplied to pharmaceutical users within the region.

Meanwhile, in the event of a disaster, the management and sorting of pharmaceuticals may become challenging owing to the accumulation of a large quantity of pharmaceuticals at disaster base pharmacies and personnel shortages. In addition, even when the pharmaceutical needs that are occurring at each evacuation shelter in the affected area are unclear, the pharmaceuticals that are to be supplied to the evacuation shelters must be sorted, and the possibility of it requiring a very long time for the necessary pharmaceuticals to reach the user can also be considered. Therefore, when supplying pharmaceuticals to disaster-affected areas, The type of pharmaceuticals to be supplied from disaster base pharmacies to evacuation shelters and the extent of supply should be considered.

In this study, we assumed an earthquake to have occurred owing to the Ochigata fault zone and clarified the number of pharmaceutical users who would evacuate to each evacuation shelter. The top three types of pharmaceuticals in Hakui City with the highest number of users were used as examples (other antihypertensives users: 2849, other agents for hyperlipidemia agents users: 2465, and coronary vasodilator users: 2255). In this study, we assumed that people would evacuate to the closest evacuation shelter from each house in Hakui City, and we performed evacuation estimation based on this assumption.

**Figure 8:**
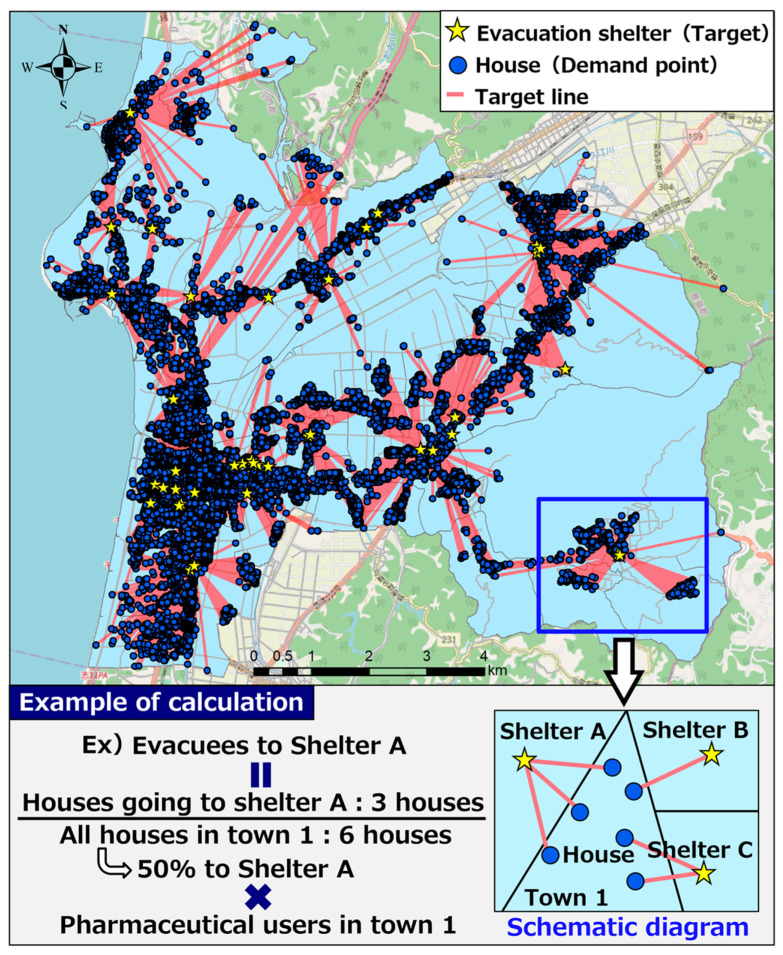
Diagram of evacuation estimation for pharmaceutical users.

Figure 8 shows a diagram of the evacuation estimation for pharmaceutical users. First, the location of evacuation shelters and houses in Hakui was examined. The National Land Numerical Data Download Site [27] was used to obtain the location information of shelters, and the center of gravity of the figure obtained from the “building perimeter line” of the Fundamental Geospatial Data Site [28] was used to obtain the location information of houses. Consequently, we obtained 40 evacuation shelters, which we selected as the target evacuation shelters for this study. Many of the shelters in Hakui are provided with cot beds, stockpiled food, and other facilities.

Next, using the aforementioned location information on shelters and houses, the nearest shelter from each house was detected. For the detection of shelters, ArcGIS Network Analyst “Nearest Facility Detection Analysis” [29] was used. “Closest facility detection analysis” measured the cost of moving between a demand point (house) and a target (evacuation shelter) and detected the target with the lowest cost. In this study, the distance of the route from the house to the evacuation shelter was used as the cost, and the closest evacuation shelter was detected. As indicated by the red line (target line) in Figure 8, the detection results enabled the spatial identification of the closest evacuation shelter calculated based on route information from each house. The shortest point from the demand point to the target is calculated based on the Dijkstra method, taking into account the road distance.

The above-mentioned detection results were used to estimate the number of pharmaceutical users who would evacuate to each evacuation shelter. As shown in the calculation example of evacuation estimation in Figure 8, the calculation method for estimation involved multiplying the proportion of the number of houses going to each evacuation shelter to the total number of houses in each town district by the number of pharmaceutical users existing in each town district to calculate the number of pharmaceutical users who evacuated to each evacuation shelter.

### 5.2. Estimation Results of Pharmaceutical Needs at Evacuation Shelters in Hakui City

The calculation results of Section 5.1 were used to calculate the number of people using other antihypertensives, other agents for hyperlipidemia agents, and coronary vasodilators who evacuated to each evacuation shelter in Hakui City.

**Figure 9:**
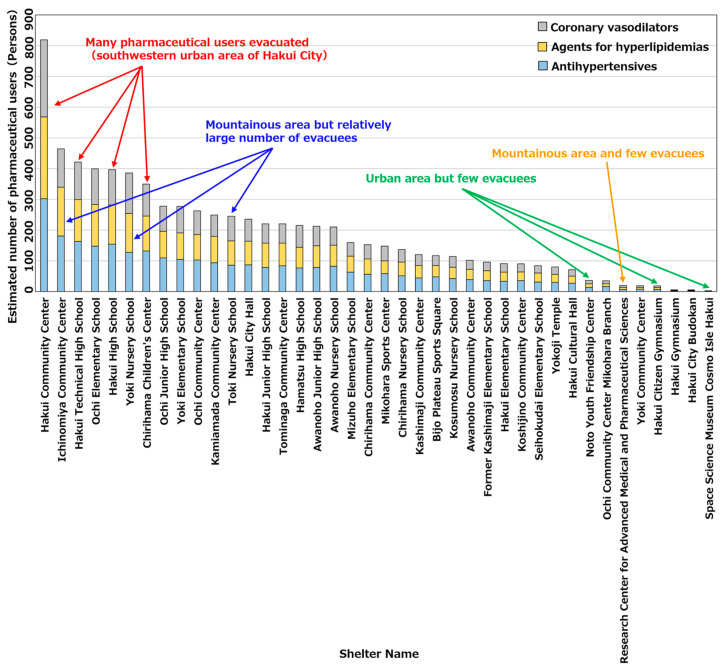
Number of pharmaceutical users by pharmaceutical type at each evacuation shelter.

Figure 9 shows the number of pharmaceutical users for each type of pharmaceutical at each evacuation shelter. As evident, there were numerous pharmaceutical users evacuating to evacuation shelters such as Hakui City Hall and Hakui Technical High School, which were located in the southwestern urban area of Hakui City; thus, there was a high need for pharmaceuticals. Meanwhile, among the evacuation shelters located in the central urban area, Hakui City Gymnasium and Cosmo Isle Hakui had the lowest number of pharmaceutical users evacuating to evacuation shelters. This is attributed to the evacuation shelters being densely packed in the central urban area; thus, the number of evacuees per location decreased. In addition, the number of pharmaceutical users evacuating to evacuation shelters located in the mountainous areas in the eastern part of Hakui City was small when compared to those in urban areas. Meanwhile, among the evacuation shelters located in mountainous areas, Ichinomiya Community Center and Yoki Nursery School had the highest number of pharmaceutical users who evacuated to evacuation shelters. This is considered to be owing to the fewer evacuation shelters in mountainous areas; thus, the number of evacuees per location increased.

### 5.3. Changes in Estimated Number of Pharmaceutical Users Due to Change in Evacuation Rate

**Figure 10:**
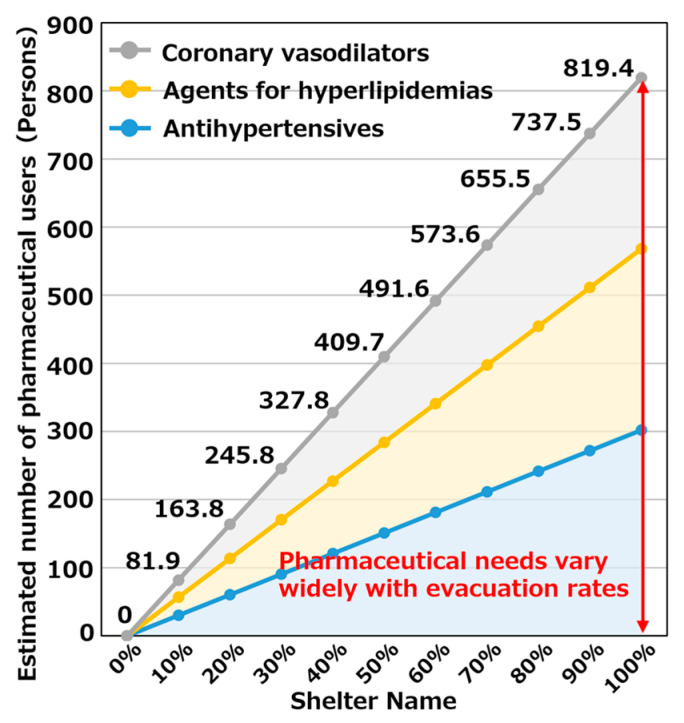
Estimation results of the number of pharmaceutical users when changing evacuation rate.

It is believed that the feasibility of evacuation may differ in the event of an actual disaster depending on the scale of the damage and the individual’s intention. We targeted the Hakui Community Center, which is expected to have the largest number of pharmaceutical users evacuating to evacuation shelters in Hakui City. For 2849 other antihypertensive agent users, 2465 hyperlipidemic agent users, and 2255 coronary vasodilator users, we considered an evacuation rate of 100% if everyone evacuated and 0% if no one evacuated. We determined the number of pharmaceutical users in evacuation shelters when the evacuation rate was varied as 0–100%. Figure 10 shows the estimation results of the number of pharmaceutical users when the evacuation rate was changed. As evident, when the evacuation rate changed, the number of pharmaceutical users changed linearly. This is because the change in the number of pharmaceutical users was estimated by multiplying the number of pharmaceutical users evacuating to evacuation shelters estimated in Section 5.2 by each evacuation rate.

Further, the range of the change in the number of pharmaceutical users was 0–819 people, which indicated that the demand for pharmaceuticals changed greatly depending on changes in the evacuation rate.

**Figure 11:**
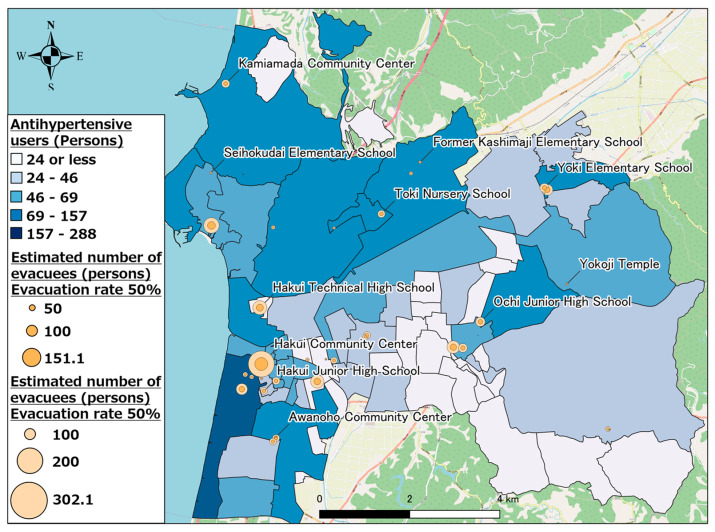
Other antihypertensive agent users (evacuation rates of 50% and 100%).

We attempted to spatially understand the changes in pharmaceutical needs that occurred at each evacuation shelter with change in the evacuation rate by visualizing changes in the number of pharmaceutical users at each evacuation shelter. Figure 11 shows the calculation results for other antihypertensive agent users when the evacuation rate was 50 and 100%. As evident, evacuation shelters such as the Hakui Community Center, where several evacuees were expected, exhibited a large change in the number of pharmaceutical users when the evacuation rate changed; whereas, in evacuation shelters such as Mikohara Annex, where a small number of evacuees were expected, there was minimal change in the number of pharmaceutical users when the evacuation rate changed. Based on the above results, evacuation shelters where a large number of evacuees are expected will exhibit a large change in pharmaceutical demand depending on changes in the evacuation rate; thus, it is concluded that the scale of the anticipated disaster and disaster situation must be focused upon, and a stockpiling and supply system for pharmaceuticals should be prepared.

## 6. Conclusions and Future Tasks

This study clarified the status of pharmaceutical prescriptions during normal times in Hakui City, Ishikawa Prefecture, to establish a prompt pharmaceutical support system that responds to the needs of an affected area in case of a disaster. We also estimated the number of pharmaceutical users (other antihypertensive agent, other hyperlipidemic agent, and coronary vasodilator users) who would evacuate to each evacuation shelter when chronic pharmaceutical users in the region evacuated owing to an earthquake disaster.

The results of the pharmaceutical prescription situation in Hakui City showed that there was a particularly high number of pharmaceutical prescriptions for lifestyle-related illnesses such as hypertension and hyperlipidemia (e.g., antihypertensive agents, hyperlipidemic agents, peptic ulcer agents) and that a high demand could be expected.

The results of estimating the number of pharmaceutical users who evacuated to evacuation shelters showed that there were numerous pharmaceutical users evacuating to evacuation shelters in regions where there are many pharmaceutical users, such as Hakui City Hall, as well as to evacuation shelters located in regions where evacuation from a wide area was expected, such as Ichinomiya Community Center, which in turn would increase the need for pharmaceuticals.

Regarding changes in the estimated number of pharmaceutical users owing to changes in the evacuation rate, evacuation shelters such as the Hakui Community Center, where a large number of evacuees is anticipated, are expected to have a large change in the number of pharmaceutical users when the evacuation rate changes; thus, there is a need to pay attention to the scale and circumstances of the anticipated disaster and prepare a pharmaceutical stockpiling and supply system.

In this study, we estimated the number of pharmaceutical users at each evacuation shelter; however, we could not consider the amount of pharmaceuticals that would actually be required. Therefore, it is necessary to examine the frequency and quantity of drugs prescribed for each drug user, and to calculate the amount of drugs required for each individual. Also, in this study, ArcGIS Network Analyst “Nearest Facility Detection Analysis” was used to estimate the number of people using medicines who take shelter in each shelter. However, depending on the damage situation in the region at the time of a disaster, location of the residents, time of day, and other factors, it may not always be possible to evacuate to the nearest evacuation shelter, and the feasibility of individuals to evacuate may also vary. Therefore, it is necessary to utilize geographical distributions of earthquake damage (e.g., distribution of seismic intensity within a region and structure of buildings), detection of the second closest evacuation shelter and utilizing location information data, and questionnaire surveys that determine the feasibility of evacuation depending on the seismic motion, season, and time of day to make evacuation estimates that reflect more detailed assumptions about the circumstances at the time of a disaster, such as considering the behavior of residents. It is necessary to conduct evacuation estimates that consider both pharmaceutical and non-pharmaceutical users as targets, compare these estimates with the evacuation estimate results of Ishikawa Prefecture, and examine the needs at evacuation shelters.

This study calculates the pharmaceutical needs that may be required in times of disaster. On the other hand, the demand for medicines may increase during a disaster due to stress, lack of sleep, and environmental changes. Therefore, based on the results of this study, it is necessary to analyze the estimation of the demand for medicines in the event of a disaster, taking physical and environmental risks into consideration.

Moreover, the period of analysis in this study was 90 days. This is because the maximum prescription period for medicines in Japan is 90 days. However, it is quite possible that patients take a variety of drugs on a daily basis. Therefore, this study did not consider time-series fluctuations in drug use. In the future, we will consider the KDB data as long-term data and conduct an analysis that takes into account the variation in drug use by individuals. Furthermore, this study used KDB data for small cities in Japan.

Since the KDB data are owned by all local governments, the results of this study can be applied throughout Japan. However, KDB data do not exist outside of Japan, so it is necessary to investigate the structure and specifications of medical big data in other countries when applying the results of this study to other countries.

## Figures and Tables

**Table 1:** Target pharmaceuticals.

Middle Classification	Sub-Classification
Hypnotics and sedatives, Antianxietics	Barbituric acids/thiobarbituric acids and preparations, Benzodiazepin derivatives and preparations, Other hypnotics and sedatives
Antiepileptics	Hydantoin derivatives and preparations, Purimidon derivatives and preparations, Other antiepileptics
Antiperkinsonism agents	Amantadine and preparations, Biperiden and preparations, Other antiperkinsonism agents
Psychotropic agents	Imipramine derivatives and preparations, Chlorpromazine and preparations, Phenothiazine derivatives and preparations, Other psychotropic agents
Cardiotonics	Digitalis and preparations, Other cardiotonics
Antiarrhythmic agents	β-blocking agents, Procaine amide derivatives and preparations, Other antiarrhythmic agents
Diuretics	Thiazide derivatives and preparations, Anti-aldosterone preparations, Carbonic anhydrase inhibitors, Other diuretics
Antihypertensives	Angiotensin converting enzyme inhibitors, Methyldopa and preparations, Other antihypertensives
Vasoconstrictors	Vasoconstrictors
Vasodilators	Coronary vasodilators
Agents for hyperlipidemias	Clofibrate derivatives and preparations, Other agents for hyperlipidemias
Other cardiovascular agents	Other cardiovascular agents
Antitussives	Ephedrine/ephedra and preparations, Dextromethorphan and preparations, Other antitussives
Expectorants	Cysteine derivatives and preparations, Bromhexine and preparations, Other expectorants
Antitussives and expectorants	Codeine derivatives and preparations, Other antitussives and expectorants
Bronchodilators	Isoprenaline derivatives and preparations, Xanthine derivatives and preparations, Other bronchodilators
Gargles	Gargles
Other agents affecting respiratory organs	Other agents affecting respiratory organs
Agents for peptic ulcer	H2-recepter antagonists, Other agents for peptic ulcer
Pituitary hormone preparations	Other pituitary hormone preparations
Thyroid and para-thyroid hormone preparations	Thyroid preparations, Antithyroid preparations
Anabolic steroids and preparations	Other anabolic steroids and preparations
Adrenal hormone preparations	Cortisone derivatives and preparations, Fluorinated corticosteroids and preparations, Prednisolone derivatives and preparations, Other adrenal hormone preparations
Estrogen and gestagen preparations	Estradiol and preparations, Estriol derivatives and preparations, Synthetic gestagens and preparations, Other estrogen and gestagen preparations
Mixed hormone preparations	Mixed hormone preparations compounded of estrogen and gestrogen
Other hormone preparations	Pancreas hormone preparations, Circulatory hormone preparations, Other hormone preparations
Anticoagulants	Dicumarol derivatives and preparations, Other anticoagulants
Other agents relating to blood and body fluides	Other agents relating to blood and body fluids
Agents for treatment of gout	Allopurinol and preparations, Colchicine and preparations, Other agents of treatment of gout
Antidiabetic agents	Sufonylurea derivatives and preparations, Biguanide derivatives and preparations, Other antidiabetic agents
Other agents affecting metabolism	Adenosine and preparations, Other agents affecting metabolism
Other antiallergic agents	Other antiallergic agents

**Table 2:** An example of the KDB data.

KDB ID	Address	Medicinal Classification (Subdivision)	Pharmaceutical Information	Quantity	Frequency	Administration Route
-	-	Other Antiallergic Agents	Fexofenadine Hydrochloride Tablets 60 mg	2	1	23
-	-	Prednisolone Derivatives and Preparations	Predonine Tablets 5 mg	1.5	1	1
-	-	Other Bronchodilators	Spiropent Tablets 10 μg	2	1	6
-	-	Other Agents for Hyperlipidemias	Rosuvastatin OD Tablets 2.5 mg	1	1	17
-	-	Other Antihypertensives	Irbesartan Tablets 100 mg	2	1	46
-	-	Other Agents for Hyperlipidemias	Pitavastatin Ca Tablets 2 mg	1	12	16
-	-	Biguanide Derivatives and Preparations	Metformin Hydrochloride Tablets 500 mg MT	1	12	2
-	-	Other Psychotropic Agents	Reflex Tablets 15 mg	1	5	51
-	-	Other Antihypertensives	Azilva Tablets 20 mg	1	3	48

## Data Availability

Data sharing does not apply to this article.
